# Evaluation of feasibility, effectiveness, and sustainability of school-based physical activity “active break” interventions in pre-adolescent and adolescent students: a systematic review

**DOI:** 10.17269/s41997-022-00652-6

**Published:** 2022-06-23

**Authors:** Alice Masini, Andrea Ceciliani, Laura Dallolio, Davide Gori, Sofia Marini

**Affiliations:** 1 Department of Biomedical and Neuromotor Sciences, Unit of Hygiene, Public Health and Medical Statistics, University of Bologna, via San Giacomo 12, 40126 Bologna, Italy; 2 Department of Life Quality Studies, University of Bologna, Campus of Rimini, Corso d’Augusto 237, 47921 Rimini, Italy

**Keywords:** Adolescent, School, Health, Physically active lessons, Active breaks, Adolescent, école, santé, cours d’activité physique, pauses actives

## Abstract

**Objective:**

The present systematic review aimed to investigate the impact of school-based physical activity (PA) interventions of “active breaks” on PA levels, classroom behaviour, cognitive functions, and well-being in pre-adolescents and adolescents attending secondary and high school.

**Methods:**

In March 2021, we performed a systematic research in *CINAHL*, *Cochrane Library*, *Embase*, *MedLine*, and *PsycINFO* databases and through grey literature. Quality assessment was performed in accordance with the Cochrane Tool for Quality Assessment for RCTs and the STROBE tool for observational studies. We included studies that investigated classroom PA interventions led by teachers such as active breaks or physically active lessons on PA levels, classroom behaviour, cognitive function, and quality of life in pre-adolescent and adolescent population attending secondary and high school.

**Synthesis:**

Three studies met the inclusion criteria. Two studies showed a positive effect of active breaks on students’ classroom behaviour and quality of life. One study registered a positive effect in the increase in school PA levels; unfortunately, this effect was not found in the overall levels of PA or in the reduction of sedentary behaviour. All three studies showed the feasibility and acceptability of active breaks intervention in secondary and high school settings.

**Conclusion:**

This systematic review suggests the potential benefit of this type of intervention integrated in the secondary and high school curriculum on classroom behaviour, school PA levels, and well-being.

**Supplementary Information:**

The online version contains supplementary material available at 10.17269/s41997-022-00652-6.

## Résumé

**Objectif:**

La présente revue systématique visait à étudier l’impact des interventions d’activité physique (AP) en milieu scolaire des « pauses actives » sur les niveaux d’AP, le comportement en classe, les fonctions cognitives et le bien-être des préadolescents et des adolescents fréquentant l’école secondaire et le lycée.

**Méthodes:**

En mars 2021, nous avons effectué une recherche systématique dans les bases de données CINAHL, Cochrane Library, Embase, MedLine, PsycINFO et à travers la littérature grise. L’évaluation de la qualité a été réalisée conformément à l’outil Cochrane d’évaluation de la qualité pour les ECR et à l’outil STROBE pour les études observationnelles. Nous avons inclus les études portant sur les interventions d’AP en classe menées par les enseignants, telles que les pauses actives ou les leçons d’activité physique sur les niveaux d’AP, le comportement en classe, la fonction cognitive et la qualité de vie chez les préadolescents et les adolescents fréquentant l’école secondaire et le lycée.

**Synthèse:**

Trois études répondaient aux critères d’inclusion. Deux études ont montré un effet positif des pauses actives sur le comportement en classe et la qualité de vie des élèves. Une étude a enregistré un effet positif dans l’augmentation des niveaux d’AP à l’école, malheureusement cet effet n’a pas été trouvé dans les niveaux globaux d’AP ou dans la réduction du comportement sédentaire. Les trois études ont montré la faisabilité et l’acceptabilité de l’intervention des pauses actives dans les écoles secondaires et les lycées.

**Conclusion:**

Cette revue systématique suggère le bénéfice potentiel de ce type d’intervention intégrée dans le programme scolaire des collèges et lycées sur le comportement en classe, les niveaux d’AP à l’école et le bien-être.

## Introduction

Regular physical activity (PA) in adolescents is positively associated with physiological and psychological health benefits such as cardiorespiratory and muscular fitness, cardiometabolic health, bone health, mental health, and cognitive function improvement (2018 Physical Activity Guidelines Advisory Committee 2018; WHO 2020; Australian Government Department of Health 2019; Singh et al. 2019). Moreover, recent evidence confirms that these health benefits could be transferred into the adulthood lifestyle (WHO 2020; Australian Government Department of Health 2019).

The World Health Organization (WHO) recommends to reach at least 60 min every day of moderate to vigorous PA for children and adolescents, aged 5–17 years, to obtain the aforementioned health benefits (2018 Physical Activity Guidelines Advisory Committee 2018). The non-achievement of PA guidelines, referred to as physical inactivity, is considered at a global level the fourth leading cause of death and a pandemic problem (Kohl 3rd et al., 2012). The most recent data from a pooled analysis of 298 population-based surveys with 1.6 million participants show that, in 2016, the global prevalence of school-going adolescents aged 11–17 years not meeting the PA recommendations was 81% with no clear pattern according to income countries group. In particular in 2016, among high-income countries, the prevalence of insufficient PA in adolescents was 89.0% (87.3–90.5%) in Australia, 72.0% (68.8–75.1%) in the United States, and 88.6% (86.8–90.2%) in Italy (Guthold et al. 2020). Overall in Europe, Steene-Johannessen et al., using accelerometer data from 30 different studies, found that only a maximum of 29% of adolescents (≥ 10–18 years) were categorized as sufficiently active, with lower PA levels among girls (Steene-Johannessen et al. 2020). A significant decrease in total daily PA was observed across the transition from primary to secondary school (Chong et al. 2020). This highlights the need to increase opportunities for adolescents to be physically active and the importance of targeting this school transition period (Steene-Johannessen et al. 2020).

In 2018, the WHO launched a global action plan (WHO, 2018) targeted to obtain a 15% relative reduction in the global prevalence of physical inactivity in adolescents by 2030, also promoting school-based PA interventions and programs (WHO, 2017). Nevertheless, if this negative trend persists, the goal will not be reached by 2030.

Nowadays, the school setting continues to be considered a key environment to promote quality physical education and several PA opportunities to enhance PA participation, well-being, and a healthy lifestyle. However, so far, class time and after-school hours contribute to most of children’s sedentary time compared with school transport, morning recess, and lunch break (WHO, 2017). The school environment appears to promote prolonged sitting time and there are long periods spent in sedentary habits during class hours (Bailey et al. 2012). Another study suggests that the current school settings might not generate a sufficient amount of PA in children and adolescents (Grao-Cruces et al. 2019).

Classroom-based PA consists of interventions that incorporate PA in class time during or between lessons with the involvement of curricular teachers.

In this scenario, classroom-based PA interventions based on short periods of PA integrated into the school routine, named active breaks (ABs), and physically active lessons with academic content (PAL) have been investigated as a potential strategy to increase school time spent in PA without decrementing educational time (Grao-Cruces et al. 2019; Masini et al., 2020a; Gallè et al. 2020; Calella et al. 2020).

The efficacy of ABs and PAL has been extensively reviewed in children but not in adolescents (Daly-Smith et al. 2018; Masini et al., 2020b; Infantes-Paniagua et al. 2021). Both PAL and classroom AB interventions showed a positive effect in increasing PA levels in primary school children objectively measured (Daly-Smith et al. 2018; Masini et al., 2020a), even confirmed by a meta-analysis that indicated a consistent trend in PA levels (Masini et al., 2020b). Moreover, the Masini et al. study reported also a positive impact of ABs on classroom behaviour (time on task) and a potential benefit on cognitive functions and academic achievement (Masini et al., 2020a). In particular, a recent systematic review with meta-analysis on the effect of AB interventions on attentional outcomes found some positive acute and chronic effects, especially on selective attention (Infantes-Paniagua et al. 2021). Furthermore, the majority of the studies included in the aforementioned reviews highlighted the feasibility and the applicability of ABs in the primary school context, whereas secondary school and high school settings were less investigated (Norris et al. 2020). As suggested by Fenesi et al., integrating physical activity directly into regular school classrooms is a promising area of research that is not yet extended to pre-adolescent and adolescent groups, but fundamental given the insufficient engagement in regular PA among adolescents (Fenesi et al. 2022).

It could be hypothesized that ABs and PAL can have the same positive effects also in adolescents. However, existing studies on school-based PA programs settled in secondary school children used several heterogeneous strategies for increasing PA during school time, such as more physical education hours or multiple interventions with organized sport sessions and extracurricular activities (Norris et al. 2020; Lonsdale et al. 2013; Cale and Harris, 2006).

Under this scenario, the present systematic review aimed to investigate the impact of school-based PA interventions based on “active breaks” on PA levels, classroom behaviour, cognitive functions, and well-being in pre-adolescent and adolescent secondary and high school children.

To our knowledge, this was the first systematic review focused only on this target group.

## Methods

The systematic review protocol was previously registered in the International Prospective Register of Systematic Reviews (PROSPERO; registration no. CRD42021230812 available from https://www.crd.york.ac.uk/prospero/display_record.php?RecordID=230812). We conducted this systematic review following the Preferred Reporting Items for Systematic Reviews and Meta-analyses (PRISMA) guidelines (Moher et al. 2009). In March 2021, a systematic research was conducted in the following electronic databases: *CINAHL*, *Cochrane Library*, *Embase*, *MedLine*, *PsycINFO.*

### Data search and search strategy

Specific criteria to define the research were applied in all the databases: we included Clinical Trial, Clinical Study, quasi experimental study, Randomized Controlled Trial with Full English text available published in the last 10 years, as this type of intervention is fairly recent, with an age range of human population from pre-adolescents to adolescents.

The following PICO (Participants, Interventions, Comparators, and Outcomes) question was developed, addressing the primary search objective, through the following search terms:

P: pre-adolescents and adolescents attending secondary and high school; I: Classroom PA intervention, active breaks intervention or physically active lessons led by the teachers within class or between class; C: theoretical lesson about PA or no PA intervention; O: PA levels, classroom behaviour, cognitive function and quality of life.

Based on the PICO, we developed different search strategies adapted for different databases using keywords terms and Boolean operators in order to be as sensitive and specific as possible (Supplementary files).

Grey literature search and hand search of other papers in key conference proceedings, journals, professional organizations’ websites, and guideline clearing houses were conducted in order to retrieve other potential pertinent studies. Additionally, we manually searched the reference lists of included studies and relevant systematic reviews to identify potentially eligible papers, not captured by the electronic searches, in accordance with the snowball technique (Greenhalgh and Peacock, 2005). Finally, journals in which included articles were published were screened to search other possible added studies.

Two independent and blind researchers (AM, SM) screened titles and abstracts and selected the eligible articles based on the inclusion and exclusion criteria. At this stage, studies were classified as “included”, “excluded”, or “undecided”. The researchers, in case of doubts about the pertinence, analyzed together the full-text articles and contacted study authors by email.

Disagreements regarding the eligibility of the studies for inclusion were resolved by discussion with the other blinded member of the researcher group. Full-text articles not included and the reasons for exclusion were recorded.

### Quality assessment and data extraction

The full-text included studies were assessed for the risk of bias, independently and blindly by the same independent researchers using “Cochrane Tool for Quality Assessment” for randomized controlled trials (RCTs) (Cale and Harris, 2006) and the “Strengthening the Reporting of Observational Studies in Epidemiology (STROBE) tool” for observational studies (Sterne et al. 2019). Any reviewers’ disagreement, upon the quality scores, was resolved through discussion with a third blind reviewer (DG) who was involved as tiebreaker. The Cochrane risk-of-bias assessment was performed for (1) random sequence generation and (2) allocation concealment (regarding bias of selection and allocation), (3) selective reporting for reporting bias, (4) blinding of participants and personal (performance bias due to knowledge of the allocated intervention), (5) blinding of outcome assessment for detection bias, (6) incomplete outcomes data for bias in attrition, and another category (7) named “other bias” based on the probable bias not covered in the other domains. We assessed risk of bias for each criterion as low, unclear (when the authors did not provide enough evidence about the bias category), and high risk. Researchers used a score to convert the Cochrane risk-of-bias tool to AHRQ (Agency for Healthcare Research and Quality) standards (Good, Fair, and Poor). The STROBE statement is a 22-item tool used for observational studies divided in three different checklists for cross-sectional, cohort, and case report studies. Based on a previous study, we adopted a cut-off for three levels of score: 0–14 poor quality, 15–25 intermediate quality, and 26–33 good quality (von Elm et al. 2014).

The data of the included papers were extracted by AM and SM using a pre-tested data extraction form. The following descriptive information were extracted from included articles: authors, country, study design, sample (number, age), intervention (type of classroom-based PA, time, duration, frequency, intensity), outcomes (instruments used), and results stratified by different outcomes. We contacted investigators and relevant study authors, seeking information about unpublished or incomplete studies.

The extracted data were then independently reviewed by DG, AC, and LD, resolving discrepancies through face-to-face discussions.

## Results

### Identification of studies

**Fig. 1:**
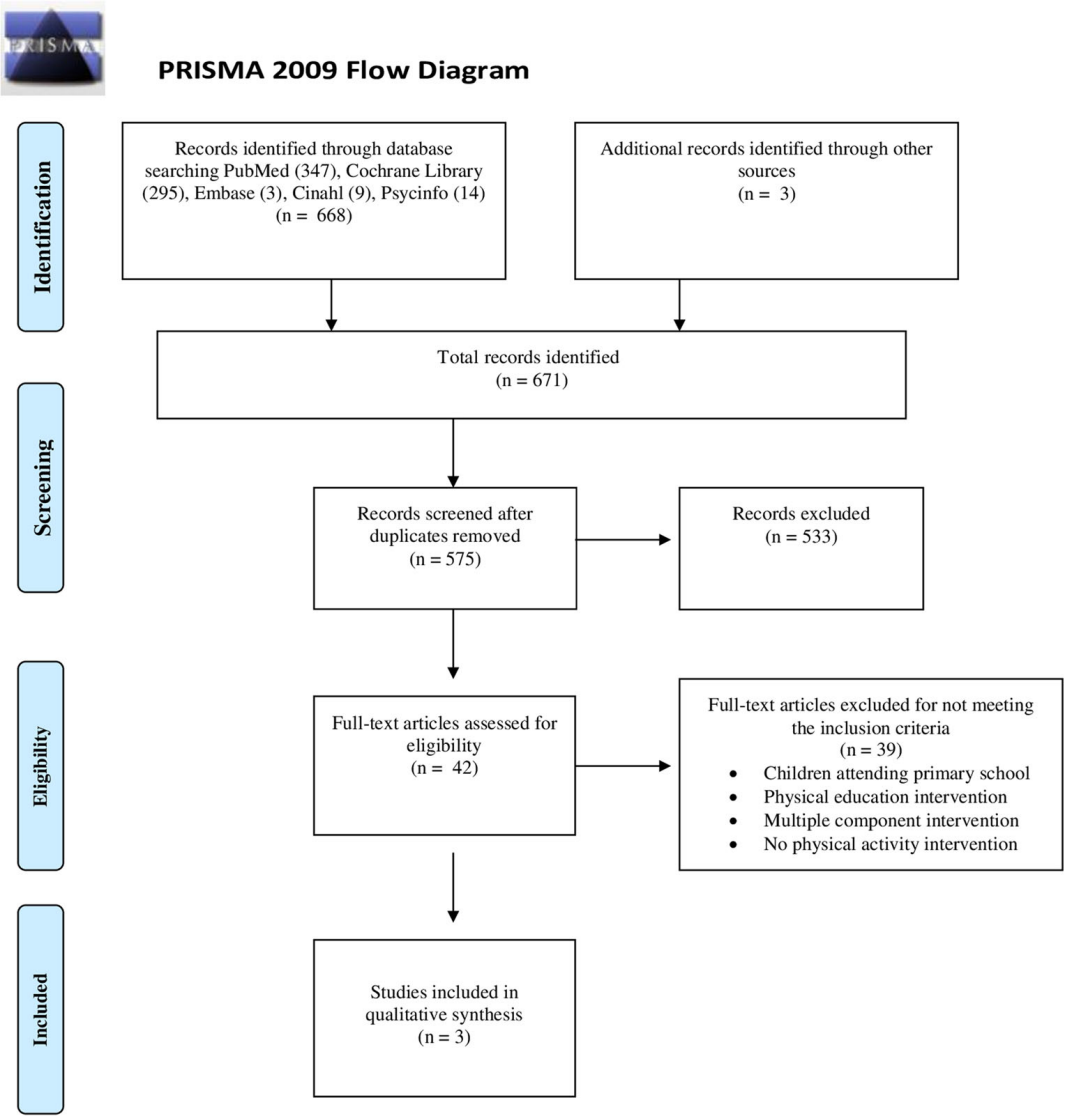
PRISMA diagram of the selection of studies

The database search and hand search retrieved 575 articles (Fig. 1). In total, 533 papers were excluded based on title and abstract. After that, a further 39 articles were removed due to not meeting the eligibility criteria described in Table 1. In the end, 3 articles fully meeting the eligibility criteria were included in the systematic review (Fig. 1).

**Table 1:** Inclusion/exclusion criteria based on PICO

Parameter	Inclusion criteria	Exclusion criteria
Population	Pre-adolescent or adolescent healthy students Aged 12–18 attending secondary school or high school	Primary school children aged 6–12, preschooler children aged 3–6, adult, workers
Intervention	Classroom PA intervention of short bouts of exercise led by teachers in class or outside class during or between academic lessons: ABs, PAL	Intervention inside physical education classes, Multiple intervention strategy (PA, diet, peer education, training lessons) Extra-school physical activity Sport practices
Comparator	No classroom PA-based intervention	Participants receiving different classroom PA intervention
Outcome	Objectively reported PA level or physical fitness; or cognitive functions or classroom behaviours or quality of life or well-being	Other outcomes
Study design	Experimental or quasi-experimental study with original primary data and full-text studies written in English	Study protocol or studies that do not present “pre” and “post” results or other papers without original data (e.g., reviews, letters to editors, trial registrations, proposals for protocols, editorials, book chapters, conference abstracts)

*PA*, physical activity; *ABs*, active breaks; *PAL*, physically active lessons

Specifically, the four main causes of exclusion were the study population attending primary school (*n*=110); the PA interventions settled inside physical education scheduled lessons (*n*=109); studies focused on multiple interventions including PA, physical education, nutrition, and behavioural component (*n*=150), and interventions without PA (*n*=109). The remaining *n*=55 studies were eliminated for having carried out the intervention outside school time or through expert trainers that organized sport and PA sessions.

### Studies characteristics

The geographic origin of the studies was *n=*1 Norway (Schmidt et al. 2020), *n*=1 United Kingdom (Gammon et al. 2019), and *n*=1 Australia (Mavilidi et al. 2021). Two studies (Gammon et al. 2019; Mavilidi et al. 2021) were designed as RCT and one study (Schmidt et al. 2020) was a quasi-experimental RCT.

Study characteristics were heterogeneous in consideration of sample size and study duration. Table 2 shows the main characteristics and results of the included studies. The sample size varied from 97 to 447 and age ranged from 13 to 16 years. The PA intervention ranged from 14 to 60 min with a frequency from 2 to 6 times per week and the study duration varied from 11 weeks to 12 months. In all three studies, the control group continued with usual normal curriculum school lessons (Schmidt et al. 2020; Gammon et al. 2019; Mavilidi et al. 2021).

**Table 2:** Characteristics of the studies included in the review

Study	Study design	Sample	Intervention	Outcomes	Results
Schmidt et al. 2020 Norwegian	Quasi-experimental study	**EG**: 197 **CG**: 447 **Age**: 13.2 (0.0)	Physically active lessons Intensity (PAL): 135 min/week + active breaks (ABs) 25 min/week Duration: 7 months	PA levels measured by accelerometers	School time total PA (min/day) EG: T0=492 (13.1) T1=505 (20.1) Time *p*: ns CG: T0=466 (9.8) T1=471 (15.6) Time *p*: ns Time*group *p*<0.000 School time sedentary time (min/day) EG: T0=182 (2.6) T1=175 (3.2) Time *p*: ns CG: T0=190 (2.2) T1=177 (2.4) Time *p*<0.001 Time*group *p*<0.001 School time daily moderate to vigorous physical activity (MVPA) (min/day)EG: T0=17 (0.8) T1=17 (10.8) Time *p*: ns CG: T0=16 (0.6) T1=13 (0.5) Time *p*<0.001 Time*group *p*<0.000
				Cardiorespiratory fitness (CRF)	**CRF** (m) **EG:** T0=1005 (8.1) T1=1021 (7.3) Time *p*<0.001 **CG:** T0=990 (6.6) T1=999 (5.3) Time *p*<0.001 Time*group *p*<0.03
				Strength	**Strength (cm)** **EG:** T0=163 (1.8) T1=171 (1.8) Time *p*<0.001 **CG:** T0=161 (1.3) T1=167 (1.4) Time *p*<0.001 Time*group *p*<0.000
				Health-related Quality of Life (HRQoL) and well-being measured by KEEDSCREEN questionnaire Higher scores indicate better HRQoL	**Physical HRQOL** **EG**: T0=46 (0.6) T1=47 (0.7) Time *p*: ns **CG:** T0=46.3 (0.5) T1=46.1 (0.5) Time *p*: ns Time*group *p*<0.01 **Psychological HRQOL** **EG:** T0=51 (0.6) T1=51 (0.8) Time *p*: ns **CG:** T0=50.3 (0.5) T1=49.0 (0.6) Time *p*<0.02 Time*group *p*<0.001 **Autonomy** **EG:** T0=53 (0.7) T1=55 (0.8) Time *p*<0.001 **CG:** T0=53.3 (0.5) T1=54.7 (0.6) Time *p*<0.02 Time*group *p*<0.004 **Peers and social** **EG**: T0=51 (0.7) T1=50 (0.9) Time *p*: ns **CG**: T0=51.2 (0.5) T1=50 (0.5) Time *p*<0.03 Time*group *P*:ns **School** **EG**: T0=53 (0.7) T1=53 (0.8) Time *p*: ns **CG**: T0=51.3 (0.5) T1=49.9 (0.6) Time *p*<0.01 Time*group *p*<0.02
				Vitality (7-point Likert scale from 1=strongly disagree to 7=strongly agree)	**Vitality** **EG**: T0=4.7 (0.1) T1=4.8 (0.1) Time *p*: ns **CG**: T0=4.8 (0.0) T1=4.5 (0.1) Time *p*<0.01 Time*group *p*<0.000
Gammon et al. 2019 UK	Pilot cluster RCT	**EG**: 130 **CG**: 130 **Age**: 13.0 (1.1)	Physically active lessons Intensity (PAL) Intensity: mean of 60 min Frequency: 6/7 times per week Duration: 11 weeks	PA levels measured by accelerometers	**Sedentary activity** (min) **EG**: T0=236.4 (31.8) T1=237.7 (40.6) **CG**: T0=217.0 (32.4) T1=222.1 (36.2) Time*group *p*: ns **Light** (min) **CG**: T0=140.5 (26.0) T1=136.6 (31.9) **EG**: T0=129.0 (26.8) T1=124.8 (31.2) Time*group *p*: ns **Moderate** (min) **CG:** T0=16.2 (7.5) T1=14.2 (7.8) **EG:** T0=11.1 (6.3) T1=10.1 (6.3) Time*group *p*: ns **Vigorous** (min) **CG:** T0=5.5 (3.9) T1=4.7 (3.5) **EG**: T0=3.1 (3.0) T1=3.0 (2.9) Time*group *p*: ns
				Mental health and well-being measured by: (a) Positive and Negative Affect (PANAS) (score 1–5) (b) Child Health Utility instrument (CHU9D) (score 0.33–1.0)	No time*group effect was found
				Time on task measured by researcher’s observation during normal frontal and PAL classes	No time*group effect was found
Mavilidi et al. 2021 Australia	RCT	**EG:** 97 **CG:** 100 **Age:** 16.0 (0.5)	Active breaks (ABs) high-intensity interval training (HIIT) Intensity: 14 min Frequency: 2/week Duration: 12 months	Cardiorespiratory fitness (CRF) measured by Pacer Fitness Gram testing procedures	**CRF** No time*group effect was found
				Time-on-task behaviour (TOT) measured by Behaviour Observation of Students in Schools and the Applied Behaviour Analysis for Teachers	**TOT** **CG:** T0=61.54 (51.95 to 71.12) T1=61.34 (51.66 to 71.03) Time *p*:ns **EG:** T0=60.89 (51.56 to 70.22) T1=79.97 (70.49 to 89.45) Time *p*<0.01 Time*group *p*<0.05 **Actively engaged** **CG**: T0=32.79 (23.22 to 42.36) T1=30.79 (21.15 to 40.43) Time *p*: ns **EG:** T0=30.30 (20.97 to 39.63) T1=47.86 (38.42 to 57.31) Time *p*<0.02 Time*group *p*<0.05 **Passively engaged** **CG:** T0=28.53 (16.82 to 40.23) T1=30.57 (18.80 to 42.34) Time *p*: ns **EG:** T0=30.57 (19.06 to 42.07) T1=31.78 (20.17 to 43.39) Time *p*: ns Time*group *p*: ns **Off-task behaviour** **CG:** T0=38.27 (28.71 to 47.82) T1=38.50 (28.84 to 48.16) Time *p*: ns **EG:** T0=39.39 (30.09 to 48.70) T1=20.13 (10.67 to 29.58) Time *p*<0.05 Time*group *p*<0.05 **Off-task verbal** **CG:** T0=18.28 (12.15 to 24.40) T1=16.64 (10.44 to 22.84) Time *p*: ns **EG:** T0=17.71 (11.76 to 23.67) T1=7.92 (1.87 to 13.98) Time *p*< 0.03 Time*group *p*: ns **Off-task motor** **CG:** T0=6.81 (4.50 to 9.12) T1=5.73 (3.34 to 8.12) Time *p*: ns **EG**: T0=4.96 (2.74 to 7.18) T1=3.35 (1.03 to 5.67) Time *p*: ns Time*group *p*: ns **Off-task passive** **CG:** T0=14.34 (10.06 to 18.61) T1=16.58 (12.22 to 20.94) Time *p*: ns **EG:** T0=16.72 (12.63 to 20.81) T1=7.48 (3.26 to 11.69) Time *p*<0.01 Time*group *p*<0.01
				Vitality measured by 6-item questionnaire Bostic (items were scored on a 7-point Likert scale, ranging from 1: not at all true to 7: very true)	**Vitality** **CG:** T0=3.87 (3.53 to 4.23) T1=3.76 (3.41 to 4.11) Time*group *p*: ns **EG:** T0=3.92 (3.59 to 4.26) T1=4.48 (4.15 to 4.82) Time*group *p*< 0.001

*EG*, experimental group; *CG*, control group; *RCT*, randomized controlled trial; *PA*, physical activity; *PAL*, physically active lessons; *ABs*, active breaks; *HIIT*, high-intensity interval training; *CRF*, cardiorespiratory fitness; *HRQoL*, health-related quality of life; *TOT*, time-on-task behaviour; *MVPA*, moderate to vigorous physical activity

The aim of the Gammon et al. study was to assess the acceptability, feasibility, and effectiveness of 60 min of PAL intervention with a frequency of 6–7 times per 11 weeks (Gammon et al. 2019). The PALs, consisting of academic lessons with moderate to vigorous movement, were performed both indoor and outdoor and led by teachers of curricular subjects. The efficacy of PAL intervention was assessed monitoring 1) PA levels using accelerometers and 2) mental health and well-being measured by Positive and Negative Affect Schedule (PANAS) (Thompson, 2007; Midgly et al. 2000) and the Child Health Utility instrument (CHU9D) (Furber and Segal 2015; Stevens 2008), and by observing 3) the time-on-task (TOT) behaviours measured by researchers during normal frontal and PAL classes (Hintze et al. 2002; Johnson et al. 2017).

The Gammon et al. study found no PAL intervention effect toward sedentary activity and to PA levels from light to vigorous intensity. Moreover, no effects were found for students’ activity behaviours or well-being indicators (Gammon et al. 2019).

The Schmidt et al. goal was to examine the effect of a school-based health-promoting program toward PA, physical fitness, well-being, and HRQoL in adolescents (Schmidt et al. 2020).

The strategy to increase PA in this study (Schmidt et al. 2020) was based on a modified Active Smarter Kids (Resaland et al. 2015) model using 30 min per day of PAL and 5 min per day of active breaks for a 7-month intervention period. PA levels were measured using accelerometers, cardiorespiratory fitness, and strength and were calculated using the Andersen test (Johnson et al. 2017, Resaland et al. 2015; Aadland et al. 2014; Andersen et al. 2008) and the Standing Long Jump (Castro-Piñero et al. 2010), respectively. HRQoL was calculated with KEEDSCREEN (The Kidscreen Group Europe 2006; Ravens-Sieberer et al. 2014) questionnaire, and subjective Vitality Scale (Ryan and Frederick 1997) was used to measure well-being and vitality. The Schmidt et al. study reported no effects of PAL and AB intervention on total PA levels; however, a significant difference in intervention group versus control group was discovered on school-based PA level from baseline to follow-up (Schmidt et al. 2020). Both intervention and control groups obtained a significant improvement (*p*<0.05) in HRQoL score especially for autonomy with major improvements in the intervention group. In the control group, HRQoL scores decreased in psychological, well-being, vitality, peer and social support, and school environment domains. Finally, considering cardiorespiratory fitness and strength performance, the Schmidt et al. study found a significant difference between the intervention and control groups (Schmidt et al. 2020).

The Mavilidi et al. study aimed to evaluate the acute effect of a school-based intervention on adolescent school students’ on-task behaviour, cardiorespiratory fitness, and vitality (Mavilidi et al. 2021).

Differently from the studies described above, this study investigated an active breaks intervention of high-intensity interval training sessions (HIIT) divided into four types of HIIT (Mavilidi et al. 2021): Gym-HIIT based on the combination of aerobic and strength exercises; Sport-HIIT using sports equipment; Class-HIIT with simple exercises to be performed in class; and Quick-HIIT using a Tabata protocol (20 s of PA followed by 10 s of rest). The AB intervention was designed with a duration from 8 to 20 min 2 times per week for an academic year. TOT behaviours were evaluated using Behaviour Observation of Students in Schools and the Applied Behaviour Analysis for Teachers (Alberto and Troutman 2003; Shapiro and Cole 1994). The Mavilidi et al. study used 6-item questionnaire Bostic (Bostic et al. 2000) to assess vitality and Pacer Fitness Gram testing procedures (Lang et al. 2018) for cardiorespiratory fitness (Mavilidi et al. 2021). The Mavilidi et al. study registered no significant effect after the AB intervention in the cardiovascular fitness tests but significant group-by-time results were observed for TOT behaviour, for students’ active engagement and vitality outcomes in favour of intervention group after 12 months of intervention (*p*<0.05) (Mavilidi et al. 2021). In particular, the authors found a significant effect for students’ off-task passive behaviour while off-task verbal and off-task motor behaviours did not change.

Following the descriptive analysis, we assessed the quality of each study differentiating RCTs from observational studies as described in Table 3. The Gammon et al. study and Mavilidi et al. study quality assessments were performed according to Cochrane risk-of-bias tool for RCTs (Figure 1S) (Gammon et al. 2019; Mavilidi et al. 2021). The Gammon et al. study was scored as “Poor Quality” due to the unclear explanation of participant’s allocation and failure blindness of participants criterion (item no. 5) (Gammon et al. 2019). Also, the study carried out by Mavilidi et al. did not match the blindness of participants criterion (item no. 5) and was scored as “Fair Quality” (Mavilidi et al. 2021). Blindness of participants was the main item causing a downgrade in the risk of bias assessment, but we are aware that this limitation derives from the nature of the experiment itself. Since the interventions consist in PA, it was not possible to create blindness of participants. In light of this, excluding element no. 5 in the assessment procedure and calculating an adequate quality assessment using the remaining six elements, the overall quality of the studies improves considerably. In accordance with the STROBE tool, we assessed the Schmidt et al. study quality as good (Schmidt et al. 2020).

**Table 3:** Quality assessment of RCTs and observational studies

Authors	Study design	Tool for assessment	Quality
Schmidt et al. 2020	Quasi-experimental study	STROBE	Good (27/33)
Gammon et al. 2019	RCT	Cochrane ROB Tool	Poor
Mavilidi et al. 2021	RCT	Cochrane ROB Tool	Fair

*RCT*, randomized controlled trial; *STROBE*, Strengthening the Reporting of Observational Studies in Epidemiology; *ROB*, risk of bias

## Discussion

The present systematic review aimed to analyze the impact of school-based physical activity intervention such as active break interventions or physically active lessons with academic content conducted by curricular teachers in secondary and high school children on PA levels, classroom behaviour, cognitive functions, and well-being. Most of the articles included in the preliminary databases search identified multiple interventions with PA, physical education, nutrition, and behavioural component; moreover, the majority of these studies were conducted in primary school children. For these reasons, due to very specific inclusion criteria, our findings were based on data from only three studies (Schmidt et al. 2020; Gammon et al. 2019; Mavilidi et al. 2021).

Generally, the present findings suggest that these types of classroom-based PA interventions showed a positive effect on students’ classroom behaviour and quality of life (Schmidt et al. 2020; Mavilidi et al. 2021). In consideration of PA levels, only one study registered a positive effect in the increase in school PA levels (Schmidt et al. 2020); unfortunately, this effect was not found in the overall levels of PA or in reduction of sedentary behaviour. All three studies reported a feasibility and acceptability of ABs and PAL interventions in secondary and high school setting.

Our results suggest that after performing one school year of ABs, adolescents were more actively involved during the school time and improved in terms of vitality, quality of school life and energy. These beneficial effects on classroom behaviour have been confirmed by previous systematic reviews focused on the primary school population (Masini et al., 2020b; Infantes-Paniagua et al. 2021).

Considering cardiorespiratory fitness and strength, the Schmidt et al. study registered a significant improvement after the interventions for experimental group, indicating that the intervention had sufficient intensity to enhance improvements in cardiorespiratory fitness and strength (Schmidt et al. 2020), while the Mavilidi et al. study did not report significant results in physical fitness (Mavilidi et al. 2021), in line with a recent review (Norris et al., 2019). These results should lead even more to take into account the effect of AB intervention not only on PA levels but also on cardiorespiratory fitness as a powerful marker for health (Pearson et al. 2017; Ortega et al. 2008). For this reason, future studies that will investigate school-based PA interventions should include cardio fitness as a fundamental health outcome. Only the Gammon et al. study did not report any significant effect of PAL on classroom behaviour, PA levels or well-being indicators, maybe due to the limited duration of the study, i.e., only 3 weeks of PAL interventions (Gammon et al. 2019). These findings are consistent with abundant evidence showing that the minimum duration of intervention that can result in meaningful and sustainable changes in the school setting is 12 weeks (Dobbins et al. 2013).

The present systematic review intended to reduce a gap in existing literature with respect to school-based AB and PAL interventions in secondary and high school students. The majority of the literature on AB and PAL interventions was focused on primary school children (Daly-Smith et al. 2018; Masini et al., 2020a; Norris et al. 2020) or combined the age groups from kindergarten to high school (Infantes-Paniagua et al. 2021).

A recent systematic review (Norris et al. 2020) emphasized the need to perform school-based PA studies in secondary and high school PA to take into account the increasing trend of sedentary lifestyle during the transition from primary to secondary school, as confirmed by Pearson et al. (2017).

Although the results should be interpreted with caution due to the limited number of studies heterogeneous in the study sample and the duration of intervention, it emerges that short physical ABs could improve students’ on-task behaviours (Mavilidi et al. 2021) and their vitality and well-being (Schmidt et al. 2020; Mavilidi et al. 2021).

Moreover, a positive effect is highlighted concerning the increase in school PA levels.

The Schmidt et al. study suggested that school-based PA interventions led to more engagement in MVPA during school hours, in contrast with the general decline in time spent in PA from primary to secondary school (Schmidt et al. 2020); however, no effects were found for the total levels of PA both by the Gammon et al. and the Schmidt et al. studies (Gammon et al. 2019; Schmidt et al. 2020) as instead was shown for the same interventions in primary school children samples (Masini et al., 2020b, Gallè et al., 2020). These results are in line with other literature that found no positive effect of school-based PA across the full day (Love et al. 2019) but an effect considering the school levels of PA (Norris et al. 2020).

Moreover, these results are consistent with a Cochrane systematic review that investigated the effectiveness of school-based interventions in increasing MVPA. The authors conclude that multi-component interventions addressing the whole school environment and incorporating PA throughout the school day (i.e., physically active lessons, physical activity breaks) are those that may have the strongest impact on time spent in MVPA (Neil-Sztramko et al. 2021).

As concerns the quality of the included studies, no RCTs (Gammon et al. 2019; Mavilidi et al. 2021) reported the strategy used to obtain blindness from the participants; however, in this type of study involving PA interventions, it is very difficult, if not impossible, to make the participants blind about the group to which they are assigned. This limitation caused a performance bias and made the general quality of the RCTs included in the current review on average poor. On the other hand, the quasi-experimental study was well designed and reported a good quality (Schmidt et al. 2020).

Our study presents some strengths and limitations. First of all, two out of three studies used objective measurements of physical activity and not self-reported assessment, thus giving greater relevance to the data obtained. However, the small number of studies included in the present systematic review should be considered in the final interpretation. Although two out of three studies have a very long duration, in general the heterogeneity in the intervention intensity, frequency, and sample size should be taken into account as a limit in the interpretation of the results.

This review may represent a starting point to bridge a gap in existing literature; however, more studies are needed to investigate the real effect of active breaks and physically active lessons on this age group and setting.

## Conclusion

The present systematic review could provide evidence of the potential benefits of introducing physical activity in secondary and high school curriculum through active breaks and physically active lessons in pre-adolescents and adolescents, especially with respect to classroom behaviour, vitality, and well-being. Future studies are necessary to better investigate the potential role of classroom-based PA as a strategy to reduce adolescents’ sedentary behaviour and improve their PA levels and cardiorespiratory fitness.

Our review should be the starting point for closing the gap in the effectiveness of classroom-based physical activity interventions for this age group and setting.

## Supplementary Information

ESM 1 (DOCX 53 kb)

ESM 2 (DOCX 185 kb)

ESM 3 (DOCX 67 kb)

## Data Availability

All data and materials are available by written request to the corresponding author.
