# Supplementary material for: Evaluation of feasibility, effectiveness, and sustainability of school-based physical activity “active break” interventions in pre-adolescent and adolescent students: a systematic review
Source: Can J Public Health. 2022 Jun 23;113(5):713–25. doi: 10.17269/s41997-022-00652-6 (PMC9481789; doi:10.17269/s41997-022-00652-6)
Supplement: Supplementary file 1 — (DOCX 53 kb) [file 41997_2022_652_MOESM1_ESM.docx]

**Appendix A. Supplementary file**

**Search term**

“(((Active Breaks) OR (Classroom break) OR (Active class) OR (Brain break) OR (Classroom break) OR (Classroom movement break) OR (Movement break) OR (Lesson break) OR (Physically active lesson) OR (Active lesson)) AND ((Pre-adolescent) OR (Adolescent) OR (children) OR (Child)) AND ((Secondary School) OR (Secondary Schools) OR (Middle school)) AND ((Physical Activity) OR (Exercise) OR (Exercises) OR (Physical Activity) OR (Activities Physical) OR (Activity Physical) OR (Physical Activities) OR (Exercise Physical) OR (Exercises Physical) OR (Physical Exercise) OR (Physical Exercises)))”.
