# Supplementary material for: Evaluation of feasibility, effectiveness, and sustainability of school-based physical activity “active break” interventions in pre-adolescent and adolescent students: a systematic review
Source: Can J Public Health. 2022 Jun 23;113(5):713–25. doi: 10.17269/s41997-022-00652-6 (PMC9481789; doi:10.17269/s41997-022-00652-6)
Supplement: Supplementary file 2 — (DOCX 185 kb) [file 41997_2022_652_MOESM2_ESM.docx]

**Appendix B. Supplementary File**

**Figure 1S.** Quality assessment of RCTs in accordance with the Cochrane Tool for Quality Assessment

**
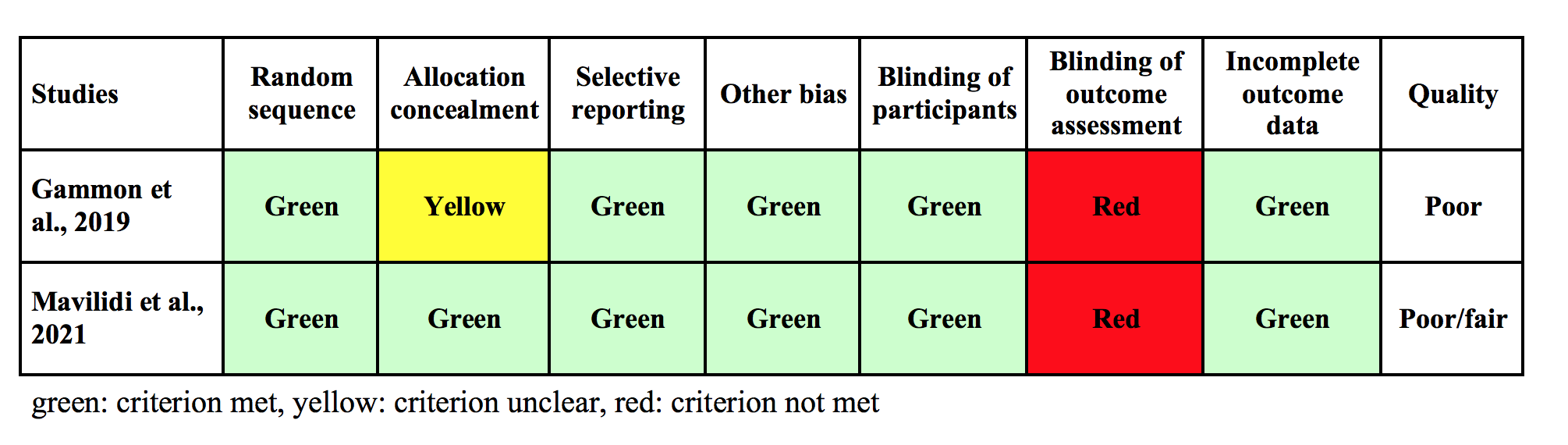
**
