# Supplementary material for: Evaluation of feasibility, effectiveness, and sustainability of school-based physical activity “active break” interventions in pre-adolescent and adolescent students: a systematic review
Source: Can J Public Health. 2022 Jun 23;113(5):713–25. doi: 10.17269/s41997-022-00652-6 (PMC9481789; doi:10.17269/s41997-022-00652-6)
Supplement: Supplementary file 3 — (DOCX 67 kb) [file 41997_2022_652_MOESM3_ESM.docx]

**Appendix B. Supplementary File**

Figure 2S Quality assessment in accordance with the STROBE

|  | Schmidt et al. 2020 |
| --- | --- |
| Title /abstract | 2 |
| Background | 1 |
| Objective | 1 |
| Setting | 1 |
| Study design | 1 |
| Participants | 1 |
| Variables | 1 |
| Data sources | 1 |
| Bias | 0 |
| Study size | 1 |
| Quantitative variables | 1 |
| Statistical method | 3 |
| Results participants | 3 |
| Descriptive data | 2 |
| Outcome data | 1 |
| Main results | 2 |
| Other analyses | 0 |
| Discussion key results | 1 |
| Limitation | 1 |
| Interpretation | 1 |
| Generalizability | 1 |
| Funding | 1 |
| Total | 27= Good |
| Results | 0-14 as poor quality, 15-25 as intermediate quality and 26-33 as good quality of the study. |
